# The Great Obstetrical Syndromes and the Human Microbiome—A New Frontier

**DOI:** 10.5041/RMMJ.10076

**Published:** 2012-04-30

**Authors:** Ido Solt, Offer Cohavy

**Affiliations:** 1 Department of Obstetrics and Gynecology, Rambam Health Care campus, Haifa, and The Rappaport Faculty of Medicine, Technion – Israel Institute of Technology, Haifa, Israel;; 2 Department of Chemistry and Biochemistry, University of California, Los Angeles, CA, USA

**Keywords:** Micro-organisms, microbiology, microbiota, microbiome, metagenome, gynecology, obstetrics

## Abstract

Over the last two decades, advanced molecular genetics technology has enabled analysis of complex microbial communities and the study of microbial genomics. Interest has grown in characterizing the microbiome, defined as a collective microbial community and its extensive genome, as a clue to disease mechanisms. “The Human Microbiome Project,” sponsored by the NIH Common Fund, was established to characterize the pathology-associated human microbiome in nasal passages, oral cavities, skin, the gastrointestinal tract, and the urogenital compartment. In particular, characterization of urogenital microbiota may elucidate etiologies of complex obstetrical syndromes and factors in fetal development that define risk for pathology in adulthood. This article summarizes recent findings defining the microbiome associated with the female urogenital compartment in child-bearing age women. We also describe our analysis of microbiome samples from the oral, vaginal, and rectal compartments in a cohort of pregnant women. Findings present technical considerations in the characterization of microbial diversity and composition associated with gestational diabetes as a model pregnancy-associated pathology.

The presence of micro-organisms in our environment and their significance in human health and disease have been known for centuries. In 1675, using his handcrafted microscope, Antonie Philips van Leeuwenhoek (1632–1723) first observed single-celled organisms. After 150 years, the animalcules, as he called them, became known as micro-organisms.^1^ Later pioneers, Pasteur (1822–1895), Cohn (1828–1898), and Koch (1843–1910), the “fathers of modern microbiology”, further established the complexity of the microbial world.

Initially, micro-organisms were studied by cultivation on nutrient-rich plates. Although yielding important information, this approach does not allow analysis of species that cannot be cultured in the laboratory. Environmental and marine microbiology studies suggest that only about 1% of the diverse microbial world can be cultivated using traditional methods, a phenomenon known as the “the great plate count anomaly.”^2^ Recently developed molecular microbiology techniques have enabled culture-independent analysis of complex microbial communities in the human body. During the last two decades, examination of micro-organisms at the molecular level, using rapid, cost-effective DNA-sequencing technology, set the foundation of modern microbiomics. This approach allows genetic identification of individual micro-organisms in a complex community but also offers a glance into the fascinating world of microbial genetics, or metagenomics when including the host genome. In 1995, the first complete genome of a free-living micro-organism, *Haemophilus influenzae* was sequenced.^3^ By 2007, more than 1,000 genes of cultivation-resistant micro-organisms were sequenced.^4^ Today, microbial genomics study tools enable the sequencing of a bacterial chromosome composed of 4,000,000 base pairs in just one day.^5^

Microbial genomics studies can provide insights into bacterial population structure, phylogenetic evolutionary history, growth requirements, protein expression, and associated immune responses. Elucidating the microbial–host interface in health and disease may enhance our understanding of virulence factors and pathological mechanisms, thus facilitating the development of powerful diagnostic tools, vaccines, and therapeutic intervention.^6^

The term microbiome was coined in 2001 by Hooper et al.^7^ A microbiome is the collective genomes of microbiota, or widely defined as the totality of micro-organisms and their genomes in a particular environment. Diverse microbiomes exist in every ecological environment, including marine and soil systems, as well as multiple interface compartments on the human body. The human microbiota contains an estimated 10^14^ micro-organisms—10 times the number of human cells in the body. The collective human microbiomal genome includes over 100 times the number of genes found in the human genome.^8^ Hattori and Taylor suggested that we should regard ourselves as “superorganisms,” inclusive of resident micro-organisms, and that the composite human–mirobiomal genome be referred to as the human “metagenome.”^8^

Sponsored by the NIH Common Fund, an international collaborative, “The Human Microbiome Project,” was launched in 2007.^9^,^10^ Its aim is to collect, integrate, and characterize the genomic sequences of microbial communities at five different sites of the human body: nasal passages, oral cavities, skin, gastrointestinal tract, and urogenital tract, and to analyze the role of the microbes in human health and disease.^9^,^10^

In order to detect microbial perturbations in association with pathology, a conserved “core” microbiome must be defined, perhaps at a species-level phylotype in a specific body habitat. In their largest human microbiota time series analysis to date, Caporaso et al. reported minor overall compositional differences among individuals for a given compartment, and that a surprisingly small yet stable temporal “core human microbiome” exists within an individual over time.^11^ They suggested a minimal core microbiome, with the complexity of the core decreasing as follows: mouth > gut > palms > across body sites within an individual > across body sites and individuals.^11^

The intestinal microbiome is currently the one most comprehensively explored.^8^ Though, of the five primary microbiomal compartments defined by “The Human Microbiome Project”, the microbiome of the urogenital tract is one of the least understood. Molecular microbial interactions at the interface between vaginal epithelia and resident microflora emerge as a “new frontier” in the study of invasive as well as non-invasive pathologies. The following section summarizes current knowledge of the resident microbiome in the female genital tract.

Currently, etiology is unknown for some of the most important obstetric conditions, such as pre-eclampsia, premature preterm rupture of membranes, premature labor, preterm delivery, intrauterine growth restriction, gestational diabetes, abruptio placentae, late abortions, stillbirth, hyperemesis gravidarum, and gestational trophoblastic disease, although a microbial role has been implicated in all these conditions. In a recent publication, Romero coined the term: “The great obstetrical syndromes,”^12^ referring to syndromes characterized by: multiple etiologies, long preclinical stage, frequent fetal involvement, often adaptive clinical manifestations, and predisposing genetic interactions.^13^ Diagnosis and treatment for any of these conditions is challenging,^13^ although changes in the microbiota were suggested to play a role.^14^

Barker hypothesized the perinatal period to determine future health and propensity for diseases.^6^,^15^ Hence, intrauterine factors affecting the fetus may also elevate risk for the development of hypertension, diabetes, stroke, coronary artery disease, and other conditions in adulthood. One pathological process implicated in multiple pathologies is intrauterine infection, and the role of micro-organisms strategically situated to affect this process should be explored. In the first in-depth study of microbial composition and ecology in the vaginal compartment of asymptomatic reproductive aged women, Ravel et al.^16^ characterized 396 healthy North American women, representing four ethnic groups: Caucasian, Afro-American, Hispanic, and Asian. Characterizing <2,000 bacteria per vaginal sample detected significant differences in dominant bacteria species as well as vaginal pH in association with ethnicity.^16^ A recent study by Taniguchi’s group extended the analysis to the complex vaginal microbiota in women with bacterial vaginosis.^17^

Dominguez-Bello et al. reported that the delivery mode, vaginal or cesarean, shapes the acquisition and structure of the initial microbiota in newborns.^18^ The relatively sterile neonate rapidly harvests micro-organisms from the environment, and much of the newborn microbiota is inoculated by his mother during and after delivery. Comparison of the microbiota of four vaginal and six cesarean born babies in Venezuela revealed that cesarean born neonates acquired microbiota closely resembling their mother’s skin microbiota, while vaginally born neonates acquired microbiota that resembled their mother’s vaginal microbiota. Regardless of delivery mode, neonates developed early bacterial communities that were undifferentiated across multiple body habitats. The effect of delivery mode on infant microbiota and its impact on development and future health should be further studied.

Preterm birth is the leading cause of neonatal morbidity and mortality worldwide. While the etiology is not fully understood, intrauterine infection may account for 25%–40% of preterm deliveries.^19^ Understanding the microbiology of the female urogenital tract, and the role that the microbiome might play in preterm deliveries, is certainly one of the “holy grails” of the search for the relationship between the microbiome and the “great obstetrical syndromes.”^20^

Our group recently formed a research platform for the study of the microbiome in human pregnancy. A biorepository of high-quality specimens was established for microbial samples of multiple anatomic sites in pregnant women. Microbial composition studies were based on molecular analysis of bacterial 16S rRNA genes as an identifier. Initial analysis demonstrated that deep 16S rRNA sequencing enhances the definition of microbial composition and diversity in the vaginal and rectal compartments during pregnancy,^21^ enhancing “resolution” of composition and diversity differences previously reported by Ravel and others.

The number of sequences acquired per specimen is inversely associated with the number of samples multiplexed in a single sequencing reaction. Therefore, our initial objective was to determine the number of 16S rRNA sequences required per sample for adequate definition of microbial communities in the oral, vaginal, and rectal compartments during pregnancy. Methodology included standard DNA extraction from swab specimens of the oral cavity, vaginal mucosa, and rectal surface of a cohort of 29 third-trimester women. Microbial identity was determined by polymerase chain reaction (PCR) amplification and sequencing of the 16S rRNA gene. We acquired 1,000–22,000 sequences per specimen (SPS), and the QIIME pipeline^22^ was used to assign sequences to the respective specimen and establish diversity parameters. Diversity in each anatomic site was defined by the number of unique operational taxonomic units (OTU >97% identity) that correlated with the simulated number of sequences obtained by pyrosequencing (Figure 2). A total of 1.3 million 16S rRNA sequences were sorted into 6,174 OTUs, representing unique genera. In the oral compartment, <2,500 sequences were required to detect the maximum of 220 genera per specimen. Microbial complexity was limited to 220 distinct genera even at a sequencing depth of 6,500 SPS, defining a diversity ceiling which is similar to the non-pregnant state. As expected, in the rectal compartment, diversity exceeded 650 genera per specimen with consistent linear increases through 6,500 SPS indicating a highly complex microbial environment. Surprisingly, in the vaginal compartment linear increases in the number of genera were also detected through the collection of >6,500 16S rRNA SPS, similar to the rectal compartment, and >400 genera were identified per specimen in 21/29 subjects (Figure 1). Microbial composition in the vaginal compartment was complex in the majority of women, exceeding 600 distinct genera, which requires a sequencing depth of 6,500 SPS in a subset of pregnant women. At a sequencing depth of 6,500 SPS, this study represents the most extensive characterization of the vaginal microbiome, since no analysis beyond 2,200 SPS is currently available for the vaginal compartment in the pregnant or non-pregnant state. We conclude that in term pregnancy, a high level of microbial composition complexity exists in the vaginal and rectal compartments, which would require deep sequencing of 16S rRNA genes to define composition and diversity.

**Figure 2:**
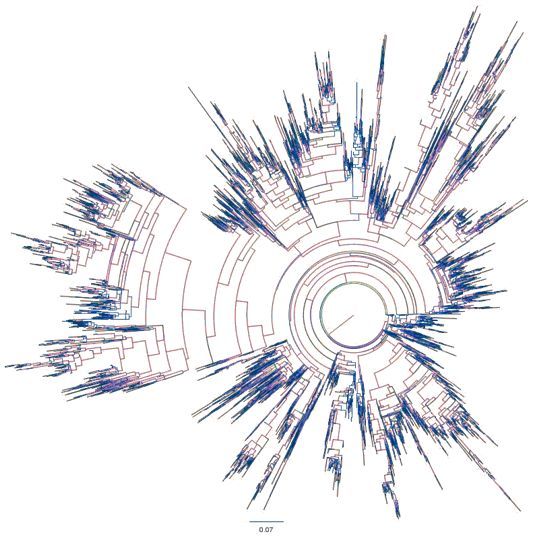
**Phylogenetic tree demonstrating successful cloning of a diverse library of microorganisms.** Microbial 16S rRNA defined a total of 6,174 OTUs (>97% identity).

**Figure 1:**
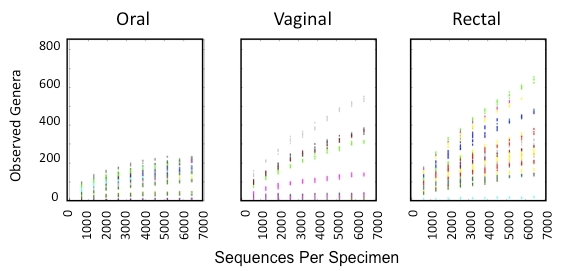
**Phylogenetic diversity and sequencing depth requirement in multiple anatomic sites.**

In a separate study, we applied these molecular methods and established diversity and composition parameters to the analysis of microbial phylogeny in the oral, vaginal, and rectal microbiomes in gestational diabetes prior to onset of active labor.^23^ Microbial 16S rRNA was amplified by PCR using conserved regions. Samples were pooled into a single pyrosequencing run. QIIME pipeline was used to validate microbial 16S rRNA sequencing quality and assign sequences to the original specimens as above. Then, a basic local alignment search tool (BLAST) was used to identify OTU sequence homology in the bacterial databases of the National Center for Biotechnology Information, to name specific genera and form a phylogenetic tree. Data indicate skewed prevalence for multiple genera in either gestational diabetes or the healthy state, suggesting changes in microbial composition for the oral, vaginal, and rectal compartments in women with gestational diabetes when compared with healthy pregnancies (Table 1 and Figure 3).

**Table 1:** **Skewed microbial composition in oral, vaginal, and rectal compartments in normal versus gestational diabetes pregnancies.** Frequent genera were present in greater than 25% differential of women with either normal pregnancies or gestational diabetes. Exclusive genera were present in greater than 25% of subjects in only one group.

		**Oral**	**Vaginal**	**Rectal**
Total Genera		275	112	673
Gestational Diabetes	Frequent	13	5	25
	Exclusive	5	5	3
Normal	Frequent	8	2	15
	Exclusive	4	2	8

**Figure 3:**
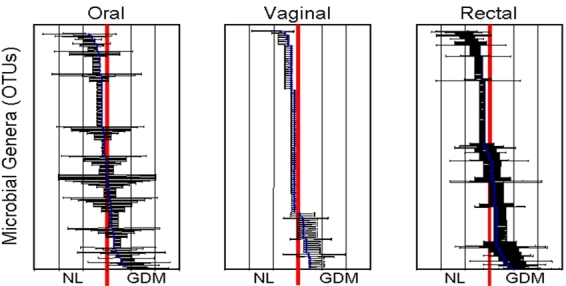
**Oral, vaginal, and rectal phylogeny in normal pregnancies (NL) and gestational diabetes melitus (GDM).**

In conclusion, the microbiome of the urogenital tract plays an important role in health and disease,^24^,^25^ prompting more comprehensive study. In particular, relationships between obstetrical pathologies and the vaginal microbiome should be defined and causality differentiated from secondary ecosystem perturbations.^23^

## Abbreviations:

OTU: operational taxonomic units;
PCR: polymerase chain reaction;
SPS: sequences per specimen.
